# *Escherichia coli* Bacteriocins: Antimicrobial Efficacy and Prevalence among Isolates from Patients with Bacteraemia

**DOI:** 10.1371/journal.pone.0028769

**Published:** 2011-12-19

**Authors:** Maruška Budič, Matija Rijavec, Živa Petkovšek, Darja Žgur-Bertok

**Affiliations:** Department of Biology, Biotechnical Faculty, University of Ljubljana, Ljubljana, Slovenia; University of Birmingham, United Kingdom

## Abstract

Bacteriocins are antimicrobial peptides generally active against bacteria closely related to the producer. *Escherichia coli* produces two types of bacteriocins, colicins and microcins. The *in vitro* efficacy of isolated colicins E1, E6, E7, K and M, was assessed against *Escherichia coli* strains from patients with bacteraemia of urinary tract origin. Colicin E7 was most effective, as only 13% of the tested strains were resistant. On the other hand, 32%, 33%, 43% and 53% of the tested strains exhibited resistance to colicins E6, K, M and E1. Moreover, the inhibitory activity of individual colicins E1, E6, E7, K and M and combinations of colicins K, M, E7 and E1, E6, E7, K, M were followed in liquid broth for 24 hours. Resistance against individual colicins developed after 9 hours of treatment. On the contrary, resistance development against the combined action of 5 colicins was not observed. One hundred and five *E. coli* strains from patients with bacteraemia were screened by PCR for the presence of 5 colicins and 7 microcins. Sixty-six percent of the strains encoded at least one bacteriocin, 43% one or more colicins, and 54% one or more microcins. Microcins were found to co-occur with toxins, siderophores, adhesins and with the Toll/Interleukin-1 receptor domain-containing protein involved in suppression of innate immunity, and were significantly more prevalent among strains from non-immunocompromised patients. In addition, microcins were highly prevalent among non-multidrug-resistant strains compared to multidrug-resistant strains. Our results indicate that microcins contribute to virulence of *E. coli* instigating bacteraemia of urinary tract origin.

## Introduction

Antibiotic resistance of bacterial pathogens is one of the greatest global threats to public health care. To prevent selection and dissemination of resistance, the use of traditional antibiotics must be limited and alternative effective therapies must be sought [1]. Of high potential are bacteriocins, ribosomally synthesized proteinaceous compounds that are generally active against bacteria closely related to the producer [2], [3], [4], [5].

*Escherichia coli* is known to produce two types of bacteriocins, classified by their molecular weight into colicins (25-80 kDa) and microcins (<10 kDa). Colicins and microcins are similar in many ways, but in contrast to colicins, microcin synthesis is not lethal to the producing strain and is not regulated by the DNA damage inducible SOS system. Further, almost all colicins are plasmid encoded, whereas microcin encoding genes are also found on the chromosome. Colicins act by either: (i) membrane permeabilization, (ii) nuclease activity or (iii) inhibition of peptidoglycan and lipopolysaccharide O-antigen synthesis [6]. Their activity requires binding to a specific receptor in the outer membrane and translocation through the outer membrane to the target by the Tol or TonB machinery [7]. On the other hand, microcins have been classified according to the presence, nature and localization of post-translational modifications, gene cluster organization, and leader peptide sequences. Class I microcins are peptides with a molecular mass below 5 kDa, and are subjected to extensive post-translational modifications (B17, C7 and J25). Class II microcins are higher molecular mass peptides (5-10 kDa), and are subdivided into two subclasses: class IIa microcins which may contain disulfide bonds but no further post-translational modifications (L, ColV and 24), and class IIb linear microcins that have a C-terminal posttranslational modification by the attachment of catechol of the salmochelin type (E492, H47, I47 and M) [8].

*E. coli* strains belong to the commensal intestinal flora however, particular strains are the causative agents of serious intestinal and extraintestinal infections [9]. In addition, *E. coli* strains cause postweaning diarrhea (PWD) and edema disease in swine [10]. Extraintestinal pathogenic *E. coli* strains (ExPEC) are a common cause of urinary tract infections, neonatal meningitis, osteomyelitis, pneumonia, surgical site infections, skin and soft tissue infections (SSTI) and bacteraemia. Virulence factors produced by ExPEC namely, adhesins, siderophores, toxins, such as α-hemolysin and cytotoxic necrotizing factor, as well as proteins impairing the innate immune response, such as TcpC, facilitate bacterial growth and persistence in the host [11], [12], [13], [14]. Bacterial bacteraemia and septicaemia represent a significant emerging clinical problem. More than 40% of bacteraemia cases, community and hospital acquired, are instigated by pathogenic *E. coli* strains and represent the main cause of mortality as well as a large economic burden. Most cases of *E. coli* septicaemia are secondary to urinary tract infection.

The aim of the present study was to investigate the *in vitro* inhibitory activity of several isolated colicins requiring distinct receptor/translocation systems and exhibiting different mechanisms of action namely, colicin M inhibiting peptidoglycan synthesis, E6 instigating hydrolysis of rRNA, E7 cleaving DNA and the pore formers E1 and K, against a collection of *E. coli* strains isolated from patients with bacteraemia of urinary tract origin. We also tested the efficacy of combined application of three (E7, K, M) and five colicins (E1, E6, E7, K, M), respectively. In addition, to gain further insight into the role bacteriocins play among natural *E. coli* populations, we studied their prevalence among the investigated strains as well as associations of microcins with virulence factor genes, phylogenetic group, multidrug resistance (MDR) and epidemiology. Our results showed that only combinations of colicins exhibited effective antimicrobial activity, precluding evolution of resistance and that, microcins may contribute to development of *E. coli* bacteraemia of urinary tract origin.

## Results and Discussion

### Sensitivity of uroseptic *E. coli* strains to colicins

**Figure 1:**
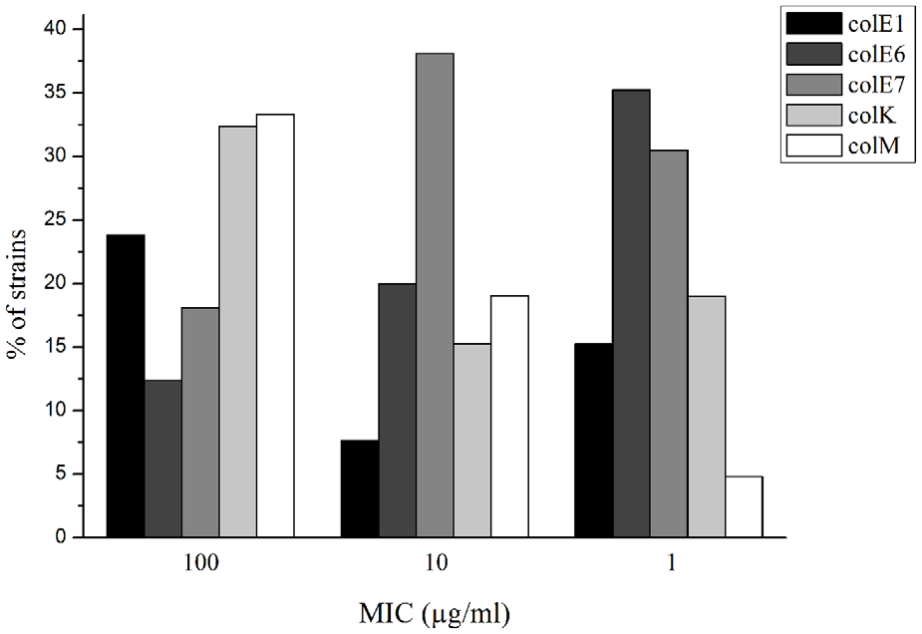
Percentages of uroseptic *E. coli* strains with minimal inhibitory concentrations (MIC) for colicins E1, E6, E7, K and M.

Testing the isolated colicins against 105 *E. coli* strains from patients with bacteraemia of urinary tract origin, revealed that colicin E7 was most effective, as 87% of the tested strains were susceptible. On the other hand, 32%, 33%, 43% and 53% of the tested strains exhibited resistance to colicins E6, K, M and E1, respectively (Fig 1). However, among susceptible *E. coli* strains various levels of susceptibility were observed (Fig 1). As noted previously, variable levels of susceptibility to colicins could be due to variability in the number of colicin receptors per cell or due to shielding of receptors by the lipopolysaccharide O-antigenic chains [15].

Colicins have been shown to be effective against *E. coli* strains associated with diarrhea including serotype O157:H7 [16], [17], [18], [19], [20], [21], [22], as well as postweaning diarrhea (PWD) and edema disease in swine [23]. Recently, dietary inclusion of colicin E1 was shown to decrease the incidence and severity of PWD caused by F18-positive enterotoxigenic *E. coli* (ETEC) [24]. Additionally, colicins have been shown to prevent colonization of urinary catheters [25] and to be effective against uropathogenic strains [26]. Colicin E1 was also shown to exhibit inhibitory activity against *Listeria monocytogenes,* the causative agent of human listeriosis in broth culture and in ready-to-eat (RTE) products [27], while colicins H and G were shown to exhibit inhibitory activity against *Salmonella* strains isolated from clinical cases [28]. Colicins are without doubt effective against pathogens nevertheless, variable susceptibility as well as resistant strains have been described [23], [26]. Due to the high prevalence of resistances against the isolated colicins we subsequently tested the efficacy of a combination of five of the selected colicins, namely E1, E6, E7, K and M. The combined action of the five colicins was effective against 98% of the tested strains as only 2% were resistant.

### Bacterial growth inhibition with colicin cocktail

The inhibitory activity of individual colicins E1, E6, E7, K and M were followed in liquid broth for 24 hours. In all experiments employing only individual colicins, an approximately five fold increase in optical density, demonstrating regrowth, was observed following 9 hours of treatment. After 24 hours of incubation, the optical densities of the cultures treated with only a single colicin were almost comparable to untreated cultures. To elucidate whether regrowth was due to loss of colicin activity or due to appearance of resistant bacterial cells, fresh colicins were added to the cultures throughout the growth cycle (every three hours). Nonetheless, an increase in optical density was detected. In addition, cells from cultures incubated for 24 hours in the presence of colicins were tested for colicin susceptibility on plates and in liquid media and were shown to exhibit resistance against the tested colicins.

**Figure 2:**
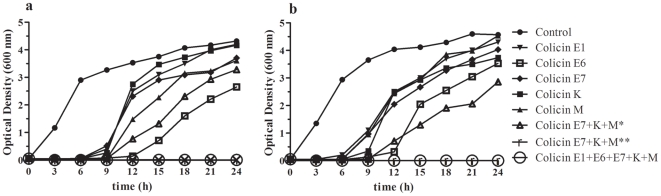
Effect of colicins on uroseptic strains. Effect of colicins E1, E6, E7, K and M on uroseptic strains UL209 (a) and UL141 (b). All colicins were added to the final concentration 1 µg/ml. Control represents the bacterial culture without colicin. * colicins were applied only at the beginning of the growth cycle; ** colicins were applied throughout the growth cycle.

Insensitivity to colicins evolves due to (i) mutations that abolish or alter a colicin receptor and (ii) due to absence of a functional system of colicin translocation. Simultaneous mutations in two or more receptors/translocation systems are far more unlikely than mutation of a single receptor/translocation system. Thus, to determine whether combinations of colicins employing different receptors, translocation systems and modes of action could provide effective antimicrobial activity, the inhibitory activity of combinations of colicins E7, K, M and E1, E6, E7, K, M was followed for 24 hours. If a combination of three colicins was employed, regrowth was observed when fresh colicins were applied only at the beginning/initiation of the growth cycle (inoculation of overnight culture cultivated without colicins into fresh media with colicins), while application every three hours throughout the growth cycle precluded regrowth. On the other hand, no resistance was observed when a mixture of five colicins was applied (Fig 2). A prerequisite for validation of the efficacy of combinations of colicins are *in vivo* experiments in model organisms.

### Prevalence of bacteriocin encoding genes among uroseptic strains

To generate further insight into the roles bacteriocins play among *E. coli* populations we subsequently analyzed the entire collection of strains from patients with bacteraemia of urinary tract origin, for one or more bacteriocins (colicins and microcins). Previous studies have suggested that colicins promote phenotypic and genotypic diversity within *E. coli* populations in the mammalian colon [29] while microcins contribute to fitness [30], and that the class IIb catechol microcins may act as potential urovirulence factors [13]. The catechol microcins take advantage of catechol receptors (Cir, Fiu or FepA) to enter *E. coli* cells. Production of catechol receptors is induced in low-iron conditions, due to which susceptible bacteria become more sensitive, providing microcin producers a competitive advantage [31]. Sixty-six percent of the strains encoded at least one bacteriocin, 43% one or more colicins, and 54% one or more microcins. Of the 75 bacteriocin-encoding strains, 43% encoded one type of bacteriocin, 45% two, 8% three, and 4% encoded four different bacteriocins. Among the investigated strains nucleotide sequences specific for colicin M were most frequent (11%), followed by sequences for colicins E1, K, E7 (8%, 6%, 2%, respectively), while colicin E6 sequences were not detected. Of the microcin specific sequences, most frequent were those encoding microcin M (35%), followed by microcin H47 (29%), microcin V (17%), microcins B17, C7 and L (3%) and microcin J25 (1%).

*E. coli* strains can be assigned to one of four major phylogenetic groups: A, B1, B2 or D [32]. Extraintestinal pathogenic *E. coli* strains mainly belong to the phylogenetic group B2 and to a lesser degree to group D [33]. Most strains encoding more than one bacteriocin belonged to the B2 phylogenetic group while bacteriocins were least frequent among group B1 strains (Table 1). The majority of microcin encoding strains (72%) belonged to the B2 group. Further, more than 85% of the strains positive for microcin H47, more than 80% strains positive for microcin M and more than 75% of the strains positive for microcin V were members of the B2 group.

**Table 1:** Frequency of *E. coli* strains encoding multiple bacteriocins with respect to phylogenetic group (groups A, B1, B2, D).

Number of encoded bacteriocins	Frequency (%)			
	A(n = 16)	B1 (n = 14)	B2 (n = 54)	D (n = 21)
No bacteriocin	56	43	11	43
One bacteriocin	25	43	28	33
Two or more bacteriocins	19	14	61	24

Analysis of bacteriocin co-occurrence revealed several statistically significant associations namely, microcins H47 and M (*P*<0.0001), microcins H47 and V (*P* = 0.0051), microcins M and V (*P* = 0.0024), microcins B17 and L (*P* = 0.0032), colicin K and microcin V (*P* = 0.0268), colicin M and microcin M (*P* = 0.0067), and colicin M and microcin H47 (*P* = 0.0417). Our data confirm the observed frequent co-association of microcins H47 and M 13,34,35 Recently, Šmajs, *et al.* [36] reported higher prevalence of colicin E1 among UTI strains compared to controls (22% and 10%) and consequently speculated that ColE1 is a potential virulence factor. In contrast, our results showed a low prevalence (12%) of colicin E1 encoding *E. coli* strains instigating bacteraemia. Moreover, Gordon and O Brien [35] showed that, among 102 bacteriocin producing commensal *E. coli* strains, 22 % produced colicin E1. The described discordances could be due to geographical variations or host characteristics [37].

### Association of microcins with virulence factors

A number of urovirulence factors (adhesins, cytotoxins, siderophores, etc.) have been identified, which appear more frequently in uropathogenic *E. coli* than in commensal or enteropathogenic strains [11], [12]. In addition, two recent studies have proposed four microcins (E429, H47, I47 and M) as potential urovirulence factors, since their production or the presence of their corresponding genes occurred predominantly in UPEC strains [30], [31]. By assay for the prevalence of *tcpC,* encoding the Toll/Interleukin-1 receptor domain-containing protein, and employing our previously published data on the prevalence of 14 virulence genes [12] among the investigated bacteraemic strains, we examined their co-occurrence with microcin encoding genes. Microcins were found to co-occur with toxins (*hly*A, *P* = 0.0014; *cnf*1, *P* = 0.0341; *usp*, *P*<0.0001), siderophores (*iro*N, *P* = 0.0029; *iro*CD, *P* = 0.0029; *fyu*A, *P* = 0.0007) adhesins (*sfa*, *P*<0.0001; *pap*C, *P* = <0.0001; *pap*G, *P* = 0.0013) and the Toll/Interleukin-1 receptor domain-containing protein (*tcpC P* = 0.0003). Of the individual investigated microcins, microcin H47 was strongly associated with toxins (*hly*A, *P* = 0.0009; *cnf*1, *P* = 0.0014; *usp*, *P*<0.0001), the *fyu*A siderophore (*P* = 0.0056), adhesins *sfa* (*P*<0.0001), *pap*C (*P* = 0.0002) and *pap*G (*P*<0.0001) as well as *tcpC* (P = 0.0002); microcin M was associated with toxins (*hly*A, *P* = 0.0001; *cnf*1, *P* = 0.0014; *usp*, *P*<0.0001), the *fyu*A siderophore (*P* = 0.0003), adhesins *sfa* (*P*<0.0001), *pap*C (*P* = 0.0002) and *pap*G (*P*<0.0001) as well as *tcpC* (<0.0001); ColV was associated with siderophores *iro*N (*P* = 0.0002) and *iro*CD (*P* = 0.0002), and adhesins *sfa* (*P* = 0.0058) and *pap*C (*P* = 0.0400); microcins B17 and L were associated with adhesin *pap*G (*P* = 0.0234). Furthermore, strains encoding two microcins, namely H47 and M, were more likely to encode toxins (*hly*A, *P* = 0.0004; *cnf*1, *P* = 0.0010; *usp*, *P*<0.0001), siderophores (*fyu*A, *P* = 0.0003; *iro*N, *P* = 0.0485; *iro*CD, *P* = 0.0485), adhesins *sfa* (*P*<0.0001), *pap*C (*P* = 0.0004) and *pap*G (*P*<0.0001) as well as *tcpC* (0.0001). Our results confirm the previous findings of Azpiroz, et al. [13], who found strong association with various virulence factors among strains producing both microcins, H47 and M. Our results indicate, on the basis of the distribution of microcin encoding genes that, microcins H47 and M may contribute to the development of bacteraemia of urinary tract origin.

Almost all bacteria require iron [38]. Previous studies have shown that a number of microcins are induced when iron is limiting and that many employ receptors for iron acquisition [8]. Thus, it has been hypothesized that microcins may be involved in competition for iron [35]. While the microcins we studied were in general shown to be associated with siderophores, additional experimental data are required to corroborate their role in competition for iron.

### Associations of microcins with immune status

Prior investigations have implied that a greater set/complement of virulence factors is required for bacterial invasion of a non-immunocompromised host than of an immunocompromised one [39], [40], [41], [42]. Our previous study showed that *usp*, *pap*C and the adhesion-encoding *sfa/foc* exhibited a statistically significant higher prevalence among strains from non-immunocompromised patients with bacteraemia of urinary tract origin [12]. To resolve whether microcin production is significant for invasion of the bloodstream, we analysed the prevalence of bacteriocins and their co-associations with virulence factors among patients with different immune status. Comparison of microcin prevalence among immunocompromised and non-immunocompromised patients revealed that their presence was significantly higher among isolates from non-immunocompromised patients (82% vs. 49%, *P* = 0.0304). Further, analysis of microcins and their co-associations with other virulence factors revealed that co-occurrences of microcins with toxins (*usp*), siderophores (*iro*N, *iro*CD, *iuc*D) and adhesins (*pap*C, *sfa*, *fim*H) were statistically significantly more frequent among strains from non-immunocompromised patients (Table 2).

**Table 2:** Significant co-associations of virulence factors and microcins in relation to host characteristics and drug resistance.

Co-association			Associated host characteristic [no. of isolates (%)]								
			Non-immunocompro-mised (n = 17)	Immunocom-promised (n = 88)	*P* value	Nosocom-ial (n = 24)	Non-nosocomial (n = 81)	*P* value	MDR (n = 22)	Non-MDR (n = 83)	*P* value
*usp*	microcins		10 (59)	19 (22)	0.006	4 (17)	25 (31)	NS	0 (0)	29 (35)	<0.001
	H47		7 (41)	17 (19)	0.062*	4 (17)	20 (25)	NS	0 (0)	24 (29)	0.003
	mcmM		8 (47)	17 (19)	0.026	4 (17)	21 (26)	NS	0 (0)	25 (30)	0.002
	V		3 (18)	4 (5)	NS	1 (4)	6 (7)	NS	0 (0)	7 (8)	NS
*hly*A	microcins		5 (29)	17 (19)	NS	5 (21)	17 (21)	NS	1 (5)	21 (25)	0.039
	H47		4 (24)	11 (13)	NS	3 (13)	12 (15)	NS	0 (0)	15 (18)	0.037
	mcmM		4 (24)	14 (16)	NS	3 (13)	15 (19)	NS	1 (5)	17 (20)	NS
	V		1 (6)	3 (3)	NS	2 (8)	2 (2)	NS	0 (0)	4 (5)	NS
*cnf*1	microcins		4 (24)	7 (8)	0.056*	2 (8)	9 (11)	NS	0 (0)	11 (13)	NS
	H47		3 (18)	6 (7)	NS	2 (8)	7 (9)	NS	0 (0)	9 (11)	NS
	mcmM		3 (18)	7 (8)	NS	2 (8)	8 (10)	NS	0 (0)	10 (12)	NS
	V		1 (6)	0 (0)	NS	0 (0)	1 (1)	NS	0 (0)	1 (1)	NS
*tcp*C	microcins		4 (24)	14 (13)	NS	0 (0)	18 (22)	0.011	0 (0)	18 (22)	0.012
	H47		3 (18)	10 (11)	NS	0 (0)	13 (16)	0.036	0 (0)	13 (16)	0.065*
	mcmM		4 (24)	13 (15)	NS	0 (0)	17 (21)	0.011	0 (0)	17 (20)	0.020
	V		1 (6)	2 (2)	NS	0 (0)	3 (4)	NS	0 (0)	3 (4)	NS
*iro*N	microcins		11 (65)	29 (33)	0.027	7 (29)	33 (41)	NS	3 (14)	37 (45)	0.0125
	H47		7 (41)	14 (16)	0.041	3 (13)	18 (22)	NS	0 (0)	21 (25)	0.006
	mcmM		8 (47)	17 (19)	0.026	3 (13)	22 (27)	NS	1 (5)	24 (29)	0.022
	V		4 (24)	13 (15)	NS	4 (17)	13 (16)	NS	1 (5)	16 (19)	NS
*iro*CD	microcins		11 (65)	29 (33)	0.027	7 (29)	33 (41)	NS	3 (14)	37 (45)	0.012
	H47		7 (41)	14 (16)	0.041	3 (13)	18 (22)	NS	0 (0)	21 (25)	0.006
	mcmM		8 (47)	17 (19)	0.026	3 (13)	22 (27)	NS	1 (5)	24 (29)	0.022
	V		4 (24)	13 (15)	NS	4 (17)	13 (16)	NS	1 (5)	16 (19)	NS
*fyu*A	microcins		12 (71)	38 (43)	0.061*	8 (33)	42 (52)	NS	3 (14)	47 (57)	<0.001
	H47		7 (41)	21 (24)	NS	4 (17)	24 (30)	NS	0 (0)	28 (34)	0.001
	mcmM		9 (53)	26 (30)	NS	4 (17)	31 (38)	NS	3 (14)	32 (39)	0.040
	V		4 (24)	11 (13)	NS	4 (17)	11 (14)	NS	0 (0)	15 (18)	0.037
*pap*C	microcins		13 (76)	32 (36)	0.003	6 (25)	39 (40)	0.060*	3 (14)	42 (51)	0.002
	H47		7 (41)	18 (20)	NS	4 (17)	21 (26)	NS	0 (0)	25 (30)	0.002
	mcmM		9 (53)	23 (26)	0.042	4 (17)	28 (35)	NS	3 (14)	29 (35)	0.069*
	V		5 (29)	9 (10)	0.049	3 (13)	11 (14)	NS	0 (0)	14 (17)	0.038
*sfa*	microcins		8 (47)	16 (18)	0.023	2 (8)	22 (27)	0.058*	0 (0)	24 (29)	0.003
	H47		8 (47)	14 (16)	0.008	2 (8)	20 (25)	NS	0 (0)	22 (27)	0.006
	mcmM		8 (47)	16 (18)	0.023	2 (8)	22 (27)	0.058*	0 (0)	24 (29)	0.003
	V		0 (0)	0 (0)	NS	0 (0)	0 (0)	NS	0 (0)	0 (0)	NS
*pap*G	microcins		3 (18)	11 (13)	NS	1 (4)	13 (16)	NS	0 (0)	14 (17)	0.038
	H47		3 (18)	9 (10)	NS	1 (4)	11 (14)	NS	0 (0)	12 (14)	0.067*
	mcmM		3 (18)	11 (13)	NS	1 (4)	13 (16)	NS	0 (0)	14 (17)	0.038
	V		0 (0)	0 (0)	NS	0 (0)	0 (0)	NS	0 (0)	0 (0)	NS
K1	microcins		4 (24)	14 (16)	NS	5 (21)	13 (16)	NS	0 (0)	18 (22)	0.012
	H47		1 (6)	5 (6)	NS	1 (4)	5 (6)	NS	0 (0)	6 (7)	NS
	mcmM		2 (12)	4 (5)	NS	1 (4)	5 (6)	NS	0 (0)	6 (7)	NS
	V		3 (18)	9 (10)	NS	4 (15)	8 (10)	NS	0 (0)	12 (14)	0.067*
*iuc*D	microcins		13 (76)	37 (42)	0.015	8 (33)	42 (52)	NS	4 (18)	46 (55)	0.002
	H47		8 (47)	20 (23)	0.068*	4 (17)	24 (30)	NS	0 (0)	28 (34)	<0.001
	mcmM		10 (59)	25 (28)	0.023	4 (17)	31 (38)	0.053	3 (14)	32 (39)	0.021
	V		4 (24)	12 (14)	NS	4 (17)	12 (15)	NS	1 (5)	15 (18)	NS
*fim*H	microcins		14 (82)	42 (48)	0.015	9 (38)	47 (58)	NS	5 (23)	51 (61)	0.002
	H47		8 (47)	22 (25)	NS	4 (17)	26 (32)	NS	0 (0)	30 (36)	<0.001
	mcmM		10 (59)	26 (30)	0.027	4 (17)	32 (40)	0.05	3 (14)	33 (40)	0.024
	V		5 (29)	13 (18)	NS	4 (17)	14 (17)	NS	1 (5)	17 (20)	NS

Fisher's exact test was used for data analysis; mcmM – microcin M; * *P*-values near the cut-off value<0.05.

A number of studies have indicated that antibiotic-susceptible and -resistant ExPEC isolates are fundamentally different bacterial populations [12], [43], [44], [45], [46], [47]. Antibiotic-susceptible strains mostly belong to the B2 phylogenetic group which are characteristically associated with higher virulence factor potential repertoire than antibiotic-resistant strains, which are typically associated with groups D and A. We therefore investigated associations between MDR (multidrug resistance) status and co-occurrence of microcins with virulence factors. Our analysis revealed that microcins were highly prevalent among non-MDR strains compared to MDR strains (63% vs. 23%, *P* = 0.0014). All 30 strains harbouring microcin H47 were non-MDR (*P* = 0.0003). In addition, 34 out of 37 strains harbouring microcin M were non-MDR (*P* = 0.0227), and 17 out of 18 ColV harbouring strains were non-MDR. Analysis of microcins and their co-associations with other virulence factors revealed that co-occurrences of microcins with toxins (*usp*, *hly*A), siderophores (*iro*N, *iro*CD, *fyu*A, *iuc*D) and adhesins (*pap*C, *sfa*, *fim*H) were statistically significantly more frequent among non-MDR strains (Table 2). Our results agree with the proposition that a decreased prevalence of virulence traits among resistant strains is a possible trade-off between resistance and virulence in ExPEC [48] and further corroborate that microcins are potential virulence factors significant in bacteraemia of urinary tract origin.

Moreover, strains from non-nosocomial infections were more likely to encode microcin and the Toll/Interleukin-1 receptor domain-containing protein (*tcpC P* = 0.011) than strains from nosocomial infections. Strains from non-nosocomial infections were also more likely to encode microcin and other virulence traits than strains from nosocomial infections however, these differences were not statistically significant (Table 2). Similar results were provided by a recent study of community-acquired, health care-associated and nosocomially acquired *E. coli* strains causing bacteraemia [47], where the authors had shown that nosocomial *E. coli* strains have reduced virulence factor content and a higher frequency of MDR.

In conclusion, our results show that application of a cocktail/mixture of three/five colicins throughout the growth cycle effectively inhibits growth of pathogenic *E. coli* strains and prevents appearance of resistance. In addition, our results indicate that microcins contribute to virulence of *Escherichia coli* instigating bacteraemia of urinary tract origin.

## Materials and Methods

### Strain collection

The *E. coli* strain collection investigated in this study has been described previously [12]. The collection consists of 105 *E. coli* strains from patients with bacteraemia of urinary tract origin, isolated at the Institute of Microbiology and Immunology, Medical Faculty, University of Ljubljana, from patients admitted to various departments of the University Medical Center in Ljubljana, from 2000 and 2001.

### Screening for colicin production

The frequency of colicin production was determined using the agar overlay method with indicator strain *E. coli* CL173 [49]. Briefly, agar plates were stab inoculated with the test strains and incubated overnight at 37°C. Colonies were lysed for 15 min using cellulose pads impregnated with chloroform. To eliminate residual chloroform vapour the plates were then exposed to air and overlaid with soft agar containing an indicator strain and incubated overnight at 37°C.

### PCR-based bacteriocin identification and prevalence of *tcpC*

Colicin producing strains were further characterized for colicin type using PCR-based screening with the primers described by Gordon, *et al.* [35].

The entire collection of uropathogenic strains was also PCR screened for genes encoding seven microcins and for *tcpC,* encoding the Toll/Interleukin-1 receptor domain-containing protein. The primers were described previously [14], [35] while microcin M specific sequences were detected with primers, mcmM F: CCTGCTATGACTGCATTCATCGACATG and mcmM R: AAACGGAAGAATGGATGATCTCGCAAA.

### Colicin isolation

To isolate purified colicin M, the *cma* activity gene was amplified together with the *cmi* immunity gene using primers ColM1 TCACTCGAGCATGGAAACCTTAACTGTTCATGCA with an added *Xho*I restriction site (underlined) and ColM2 CCACGCGTCCACTTCACAGTATGCTCACATTG with an added *Mlu*I restriction site (underlined). The PCR product was digested with restriction enzymes *Xho*I and *Mlu*I and cloned into the expression vector pET8c [50] cut with the same two restriction enzymes. Subsequently, colicin M was expressed in the *E. coli* strain BL21 (DE3) and large-scale expression was performed as previously described [51]. The colicin M containing fractions, as determined by sodium dodecyl sulfate-polyacrylamide gel electrophoresis, were dialyzed against 5 mM phosphate buffer and stored at –20°C.

Purified colicins E1 and K were isolated as described previously [23], [26] whereas for determination of inhibitory activity of colicins E6 and E7, filtered supernatants of mitomycin-C induced cells producing either colicin E6 or colicin E7 was used. Briefly, the colicin producing strains were grown in LB medium at 37°C until an OD_600_ of approximately 0.9, when colicin production was induced by addition of mitomycin-C. After harvest, the culture was centrifuged for 10 min at 17000×g, and the supernatant was stored at –80°C. Protein purity was checked by sodium dodecyl sulfate-polyacrylamide gel electrophoresis.

Protein concentration for all isolated colicins was assayed using a BCA (bicinchoninic acid) protein assay kit (Pierce).

### *In vitro* inhibitory activity of the isolated colicins

The antimicrobial efficacy of the isolated colicins was assessed against the entire collection of uropathogenic strains. To determine the minimal inhibitory concentrations (MIC) of the purified colicins, 5 µl of various concentrations (1, 10, and 100 µg/ml) of the selected colicins were spotted onto LB plates overlaid with soft agar harboring the individual investigated *E. coli* strains. Following overnight incubation at 37°C, the plates were examined for colicin sensitivity observed as clear zones of lysis of the overlaid strains. Subsequently, the inhibitory activities of colicins E1, E6, E7, and M were quantified in liquid broth. From each susceptibility group (corresponding to concentrations of 1, 10 and 100 µg/ml), two strains were chosen for the assay. Briefly, prewarmed LB was inoculated with overnight cultures of the tested strains to an OD_600_ approximately 0.05. Five ml of the inoculated LB was aliquoted into culture tubes containing either individual or combinations of three (E7, K, M) and five colicins (E1, E6, E7, K, M) at a concentration of 1 µg/ml. The tubes harboring the tested strains and colicins were incubated with shaking at 37°C and to determine the inhibitory activity of the colicins, optical density (OD_600_ values) was determined hourly.

### Statistical analysis

Co-occurrence of two bacteriocins and one of the bacteriocins with another virulence factor, co-association of bacteriocins, virulence factors and host characteristics was evaluated by using Fisher's exact test. *P*-values lower than 0.05 were regarded as statistically significant.
